# Advancing Digital Health Integration in Oncology

**DOI:** 10.2196/70316

**Published:** 2025-03-07

**Authors:** Rai Muhammad Umar Khan, Hassan Tariq

**Affiliations:** 1 Punjab Medical College Faisalabad Medical University Faisalabad Pakistan

**Keywords:** mHealth, user experience, cancer, technology acceptance model, structural equation modeling, health care app, mixed-method study, medical care, digital health care, cancer survivors, disparities, health status, behavioral intervention, clinician

The study titled “User Experience and Extended Technology Acceptance Model in Commercial Health Care App Usage Among Patients With Cancer: Mixed Methods Study” by Park et al [1] provides valuable insights into how user experience, interface satisfaction, and external motivation affect the adoption of health care apps among patients with cancer. This work aligns with long-standing theories, such as those detailed by Davis [2], emphasizing that perceived usefulness and ease of use are crucial determinants of technology acceptance. By incorporating an expanded Technology Acceptance Model, Park et al [1] shed light on several key areas where digital health solutions can be optimized for oncological care.

The importance of user-oriented design is unmistakable. Satisfaction with an interface has been repeatedly linked to greater app engagement [2], and Park et al [1] confirm that clinicians’ endorsements bolster consistent usage patterns. Gaps in digital literacy, particularly among older populations, remain a pressing concern and necessitate further policy-level interventions to promote equitable access. Yet several emerging trends suggest that further innovation and collaboration could amplify the benefits of digital health apps in oncology.

Keesara et al [3] argue that the COVID-19 crisis catalyzed a “digital revolution” in health care, unveiling the potential for remote, app-based oncology services. Harnessing these developments may require additional training and infrastructure, especially in resource-limited settings where telehealth adoption remains uneven.

In a systematic review, Kruse et al [4] highlight that both patient and provider acceptance are crucial to sustaining digital solutions. While Park et al [1] primarily focus on patient factors, encouraging provider buy-in through institutional policies and workflow integration could markedly enhance long-term adherence and improve clinical outcomes.

Ammenwerth et al [5] show that electronic patient portals can encourage patient autonomy, enabling individuals to track health metrics, communicate with clinicians, and gain personalized insights. Expanding the features of health care apps beyond simple monitoring to include robust patient portals might address the digital divide, provided adequate educational support is offered.

To conclude, Park et al [1] have laid a solid foundation for understanding how patients with cancer adopt health care apps, emphasizing the centrality of user experience and clinician endorsement. However, broader integration of telemedicine advances, joint patient-provider acceptance, and interactive patient portal functionalities could drive higher engagement rates and better patient outcomes across diverse populations. Although the work provides a basis for assessing app use among Korean cancer survivors, its generalization to other populations needs to be further investigated. Moving forward, policy makers and health care organizations should focus on closing digital literacy gaps, incorporating patient portals, and reinforcing telehealth infrastructure, thus ensuring that digital oncology solutions effectively serve patients’ needs worldwide.
